# Early response of total neoadjuvant therapy with regional deep hyperthermia for low-lying locally advanced rectal cancer: a single-center retrospective study

**DOI:** 10.1007/s00066-026-02512-5

**Published:** 2026-02-24

**Authors:** Nicole Klinge, Paul Warncke, Griseldis Petzold, Michael Schöpe, Marc Golombeck, Julia Klaus, Lutz Mirow, Hagen Rudolph, Kristin Tischendorf, Matthias Berger, Zohier Srour, Korinna Jöhrens, Mathias Hänel, Gunther Klautke

**Affiliations:** 1 Department of Radiooncology, Klinikum Chemnitz gGmbH, Chemnitz, Germany; 2 Department of Internal Medicine III, Klinikum Chemnitz gGmbH, Chemnitz, Germany; 3 Medical Campus Chemnitz, Faculty of Medicine and University Hospital Carl Gustav Carus, TUD Dresden University of Technology, Dresden, Germany; 4 Department of General and Visceral Surgery, Klinikum Chemnitz gGmbH, Chemnitz, Germany; 5 Department of General and Visceral Surgery, Zeisigwaldkliniken Bethanien, Chemnitz, Germany; 6 Department of General and Visceral Surgery, Erzgebirgsklinikum gGmbH, Zschopau, Germany; 7 Department of General, Visceral Surgery and Proctology, Diakoniekrankenhaus, Chemnitzer Land gGmbH, Hartmannsdorf, Germany; 8 Institute of Pathology, Klinikum Chemnitz gGmbH, Chemnitz, Germany

**Keywords:** Rectal cancer, Deep hyperthermia, Shincter preservation

## Abstract

**Purpose:**

Total neoadjuvant therapy (TNT) combined with regional deep hyperthermia (HT) for the treatment of patients with low-lying locally advanced rectal cancer (LARC) is a promising strategy to optimize patient outcomes. However, its feasibility in a real-world setting requires evaluation, particularly regarding sphincter preservation rates and the effects on tumor response, downstaging, and treatment.

**Methods:**

Patients with low-lying LARC who underwent TNT, including chemoradiation with regional deep HT twice weekly during chemoradiation, were evaluated with regard to sphincter preservation, downstaging, and clinical (cCR) or pathological (pCR) complete response rates. Data were obtained between 2018 and 2023.

**Results:**

A total of 49 patients with LARC at local UICC stages II (25%), III (67%), and IV (8%) were included in the analysis. The combination of TNT and deep HT demonstrated encouraging complete remission (CR) rates, with cCR or pCR achieved in 39% of patients and significant tumor downstaging in 76% of cases. A watch-and-wait concept was applied in nine cCR cases (18%). In total, 40 (82%) patients underwent surgical resection. Sphincter preservation was achieved in 31% of the patients, corresponding to a total sphincter preservation rate of 49%. Generally, TNT in combination with HT was well tolerated with an acceptable toxicity profile.

**Conclusion:**

Combining TNT with regional deep HT for low-lying LARC is a promising treatment strategy that improves tumor response rates, complete remission rates, and tumor downstaging while maintaining acceptable toxicity profiles. Further prospective randomized studies are warranted to confirm these findings and establish the role of HT in neoadjuvant protocols.

## Introduction

Among colorectal malignancies, rectal cancer accounts for around 35% of cases in the European Union and carries a high risk of local recurrence and potential postoperative complications that limit quality of life, particularly after rectal extirpation [1, 2]. To improve outcomes, total neoadjuvant therapy (TNT) has emerged as a promising approach in the management of locally advanced rectal cancer (LARC). Total neoadjuvant therapy involves the preoperative administration of both chemoradiation and consolidation or induction chemotherapy, aiming to enhance treatment outcomes by increasing pathological downstaging and addressing occult micrometastatic disease [3]. This approach has also been associated with improved pathological complete response (pCR) rates and improved disease-free survival (DFS) compared to standard neoadjuvant chemoradiotherapy (CRT; [3, 4]). The impact of TNT for LARC patients on overall survival (OS) remains debatable, since major trials showed nonsignificant OS improvements [5, 6]. The implementation of TNT has shown feasibility and effectiveness in various settings, including rural community oncology centers, where it has demonstrated higher treatment completion rates and a tendency toward improved pCR rates [7]. The sequence of CRT and consolidating chemotherapy affects local response rates, with better outcomes achieved by consolidating chemotherapy [8].

In patients with LARC characterized by Union for International Cancer Control (UICC) stage II or III tumors (and UICC stage IV with distinct oligometastasis), the response to neoadjuvant therapy is of prognostic importance. The intensity of tumor regression correlates with survival and the degree of downstaging [9–11]. In the case of low-lying lesions, tumor regression is also crucial for achieving sphincter preservation. This is accomplished through the possibility of sphincter-preserving surgery or, in cases where a clinical complete response (cCR) is achieved, by avoiding surgery altogether (watch-and-wait). It has been suggested that the rate of cCR can be increased by intensifying chemotherapy in the context of concurrent CRT through integration of oxaliplatin or irinotecan chemotherapy [12–15], increasing the time window between the end of radiotherapy and the time of surgery [16, 17]. This concept of TNT was applied in the CAO/ARO/AIO-12 trial [18]. Here, CRT with consolidation chemotherapy and subsequent surgery was superior to the induction chemotherapy arm with subsequent CRT and surgery in terms of tumor regression and the rate of CRs [18]. Apart from that, several working groups have incorporated deep hyperthermia (HT) into the RCT treatment concept, both in primary therapy and in the relapse setting with re-irradiation [19, 25].

The rationale for the use of deep HT is local enhancement of the effect of radiotherapy and chemotherapy at temperatures above 40.5 °C, aiming to achieve greater local tumor regression [20]. In LARC, the combination of deep HT and neoadjuvant RCT, usually with 5‑fluorouracil (5-FU), also led to an increase in response rates of about 10% [19, 21, 22].

In this retrospective single-center analysis, the efficacy and tolerability of HT in addition to CRT as TNT was analyzed for the treatment of low-lying LARC. The aim of this study was to determine the potential additional benefit and feasibility of deep HT in the context of TNT, especially in terms of local responses, tumor downstaging, and surgery outcomes.

## Materials and methods

### Study design and patients

The study population included patients diagnosed with low-lying LARC who received TNT treatment in combination with regional deep HT and consolidating chemotherapy at the Department of Radiooncology, Klinikum Chemnitz gGmbH between January 2018 and December 2023. Medical records of patients were screened and laboratory (toxicity), pathological examination results, and imaging findings were collected. In total, 49 patients were identified and included in the analysis. The investigation was approved by the regional ethics committee of the institution (State Chamber of Physicians of Saxony, EK-BR-20/25-1).

### Definitions

Clinical (cCR) or pathological (pCR) complete remission rates were assessed 8–12 weeks after RCT. The rate of pCR was determined by histopathological examination of the resected specimen, defined as the absence of viable cancer cells (ypT0N0 according to UICC). The rate of cCR was evaluated using proctoscopy and imaging (magnetic resonance imaging [MRI] and computed tomography [CT]). We defined cCR as the absence of visible tumor, negative biopsies, and no evidence of residual disease according to RECIST guideline [23]. All assessments followed standardized protocols and were performed by experienced investigators. Total CR rates were calculated as the sum of pCR and cCR.

### Staging and treatment

All patients underwent biopsy to confirm the diagnosis of LARC. Histological diagnosis and grading were made in accordance with the World Health Organization (WHO) classification, with immunohistochemical staining applied when necessary. After histological confirmation, all patients with rectal cancer were staged using CT of the thorax/abdomen/small pelvis, MRI of the small pelvis, and a complete colonoscopy. In most cases, the location of the tumor in relation to the anocutaneous line and the sphincter apparatus was assessed using a rigid proctoscope, otherwise the height was localized using MRI. In cases of questionable findings, the staging examinations were supplemented by MRI of the liver and/or positron emission tomography computer tomography (PET-CT).

All patients with low-lying LARC (lower tumor margin at a maximum of 6 cm from the anocutaneous line) with local UICC stage II, III, and IV (with distinct oligometastasis) treated with TNT and HT, according to a treatment regimen prospectively determined, were included in the analysis. Both TNT and HT were selected for all patients (considering contraindications) with the objective of achieving maximum remission to preserve the sphincter in accordance with institutional standard protocols and following interdisciplinary consensus.

In terms of radio- and chemotherapy, the treatment regimen corresponded to the TNT regimen from arm B of the CAO/ARO/AIO-12 study, with conventionally fractionated radiotherapy using the IMRT technique from the primary tumor containing a safety margin of 4 cm each in the direction of the aboral and oral tumor margins, and safe inclusion of the mesorectal fascia and the pelvic lymphatic drainage area with individual doses from 1.8 Gy to 50.4 Gy. Simultaneously, infusion of 5‑FU (250 mg/m^2^ days 1–14, days 22–35) and oxaliplatin (50 mg/m^2^ days 1, 8, 22, 29) were applied. This was followed by three courses of consolidating chemotherapy (oxaliplatin, 100 mg/m^2^; 2 h), leucovorin (400 mg/m^2^; 2 h), and 5‑FU (2400 mg/m^2^; 46 h repeated on day 15) before planned total mesorectal excision. The decision on further treatment strategy (watch-and-wait vs. surgery) was based on the results of restaging (cCR), the recommendations of the interdisciplinary tumor board, and the patient’s choice.

Deep HT (target temperature: 41–43 °C for 60 min) was applied with the BSD 2000 Mircowave Hyperthermia System (Pyrexar Medical; Vertrieb Dr. Sennewald Medizintechnik GmbH, Germany), a deep regional system using an annular phased-array configuration to focus thermal energy on the targeted treatment area. The identical treatment protocol was used for all patients. The treatment was planned twice a week during the CRT (concurrent targeted application of oxaliplatin), after excluding contraindications such as pacemakers, stent implantations, bypass operations, other metal implants or internal diseases. Local deep HT was applied using Sigma 60 or Sigma Ellipse ring applicators based on phased-array technology, in combination with a water-cooled bolus equipped with eight radiative dipoles. The isocenter used for HT planning was determined based on the radiotherapy planning CT, while HT planning was carried out using the device-specific planning system. The effective treatment duration was 60 min, following an additional heating phase of 20–30 min. Temperature monitoring was performed using a calibrated thermistor placed intraluminally during HT treatment. Depending on the patient’s sex, six to seven catheters were used. Temperature probes were inserted in the rectum, rima ani, vagina, inguinal region, and urinary bladder, as well as on the dorsal and ventral skin surfaces. Real-time temperature mapping was applied to monitor tissue heating. Measurements were acquired at 1‑cm intervals along the catheters over a length of up to 15 cm, with repeated measurements taken at 5‑min intervals. Rectal temperatures were calculated directly from the raw measurement data. Only data acquired during the treatment phase (thermal steady state had been achieved) were considered for analysis. The mean temperature during the treatment was calculated for each patient.

### Outcome measurements and statistics

The primary endpoint of the study was the rate of CR (pCR and cCR) after therapy. The secondary endpoint was the rate of sphincter preservation after CRT and/or surgery. Disease-free survival (DFS) was defined as the time from initiation of neoadjuvant therapy to the first documented local recurrence, distant metastasis, second primary malignancy, or death from any cause, whichever occurred first. The DFS was estimated using the Kaplan–Meier method for patients achieving cCR. Median follow-up time was calculated via the reverse Kaplan–Meier method. Adverse events according to the National Cancer Institute (NCI) Common Terminology Criteria for Adverse Events (CTCAE v.5.0) were determined based on available data from medical records. Data were analyzed descriptively using the median and range or mean and standard deviation (continuous data) and frequency distribution (categorical data). Response rates are presented as proportions with 95% confidence intervals (95% CI, Wald method). The Wald tests compared observed rates against results from the CAO/ARO/AIO-12 trial (18) as historical benchmarks (arm B). The normality of continuous variables was assessed using the Shapiro–Wilk test and Q‑Q plots. Variables meeting normality assumptions (Shapiro–Wilk *p* > 0.05 with supporting Q‑Q plot visualization) were analyzed using one-sided *t* tests. A value of *p* < 0.05 was considered statistically significant.

## Results

### Patient characteristics

From January 2018 to December 2023, 49 patients with LARC were treated with TNT and additional deep HT. In all patients the tumor was localized in the lower rectum less than 6 cm from the anal verge. Patients had a median age of 62 years (range, 22–79) at the start of RCT and more than two-thirds were male. Most patients (92%) were staged as UICC II or III. Four patients had a distant oligometastasis (UICC stage IV). These patients were considered for TNT and HT treatment based on the local disease burden and limited metastatic extent. Detailed patient characteristics are shown in Table 1.

**Table 1:** Patient characteristics.

Characteristics		Patients (*n* = 49)	Frequency (%)	Median (range)
*Median age (years)*	–			66 (22–79)
*Age (years)*	< 50	4	8	–
	50–69	34	69	
	≥ 70	11	23	
*Sex*	Female	13	27	
	Male	36	73	
*cT stage*	cT2	5	10	
	cT3	41	84	
	cT4	3	6	
*cN stage*	cN0	13	27	
	cN1	18	37	
	cN2	18	37	
*cM stage*	cM0	45	92	
	cM1	4	8	
*UICC stage*	II	12	25	
	III	33	67	
	IV	4	8	

### Treatment and toxicity

All patients included in this analysis received CRT with 5‑FU and oxaliplatin and a radiotherapy dose of 50.4 Gy analogous to the CAO/ARO/AIO-12 trial. Additionally, patients received a median of eight deep HT sessions (range, 4–10). The mean temperature at HT treatment was 40.51 °C (± 0.64 °C). Seven patients (14%) had fewer than six HT sessions. The dose density of radiotherapy and concurrent chemotherapy was not affected by deep HT. In all patients, neither dose reduction nor treatment break in radiation therapy was needed. Three patients omitted oxaliplatin in the second treatment course (day 29). The median time from RCT to surgery was 130.5 days (range, 75–231 days).

An overview of early toxicity following HT treatment is shown in Table 2. Adverse events were observed in almost all patients (98%). The most frequent side effect was radiodermatitis in 32 patients (71%), including three (6%) patients with severe (≥ grade 3) toxicities. Other severe toxicities were myocardial infarction (*n* = 1) and hepatobiliary disorder (*n* = 1). In total, HT treatment was well tolerated and showed an acceptable toxicity profile.

**Table 2:** Number of patients (and frequency) of toxic events after radiochemotherapy treatment.

Toxicity	Grade 1–2	Grade ≥ 3
Radiodermatitis	32 (65%)	3 (6%)
Proctitis	5 (10%)	0
Rectal pain	8 (16%)	0
Diarrhea	15 (31%)	0
Urinary/voiding disorders	5 (10%)	0
Cystitis	20 (41%)	0
Sensory neuropathy	15 (31%)	0
Hepatobiliary disorders	7 (14%)	1 (2%)
Myocardial infarction	0	1 (2%)
Anemia	7 (14%)	0

### Outcomes

**Fig. 1:**
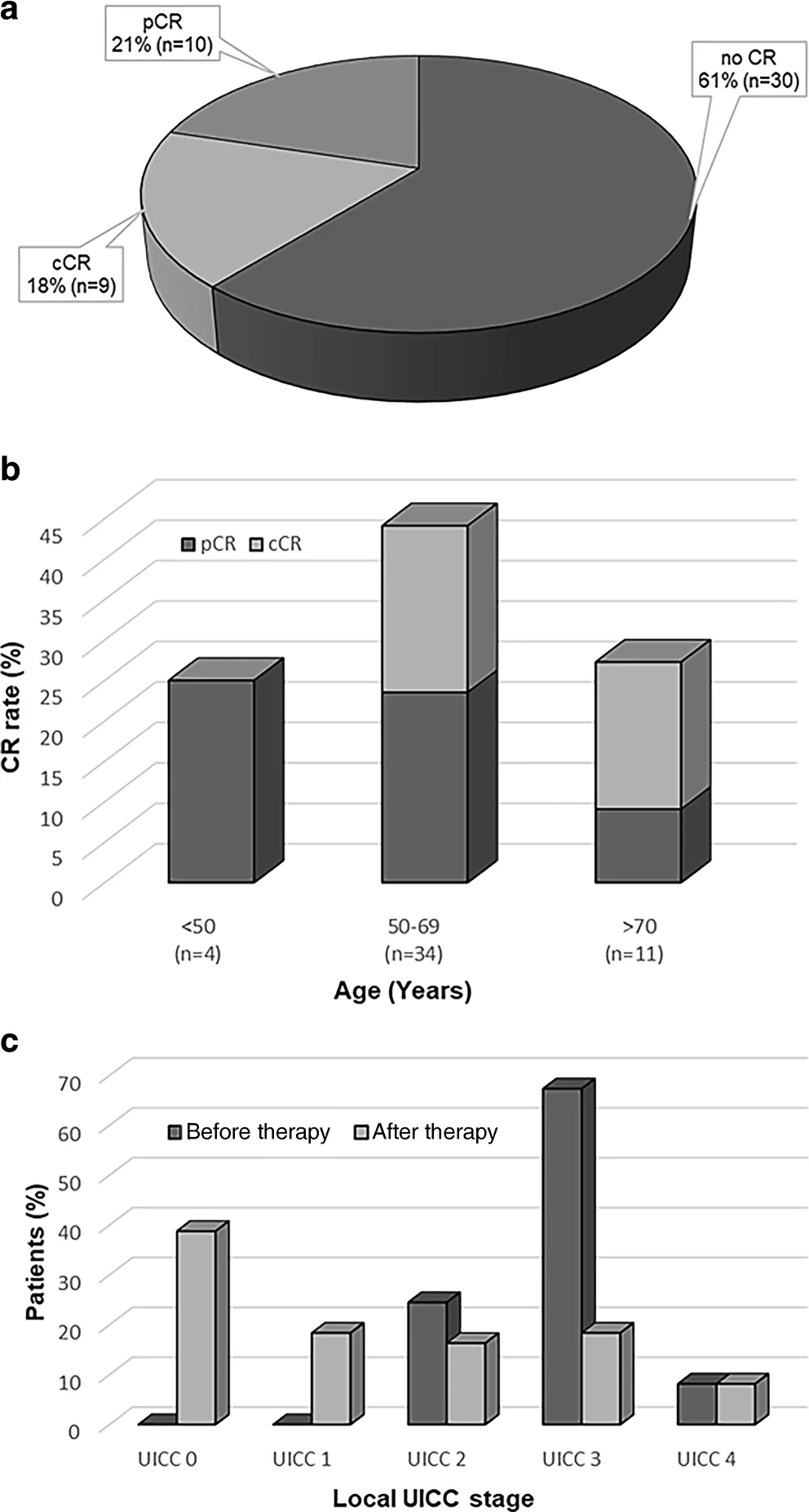
Outcome of patients after total neoadjuvant therapy and hyperthermia treatment. *cCR* clinical complete remission, *pCR* pathological complete remission, *CR* complete remission

Outcomes were assessed by pathological and clinical evaluation of remission status (Fig. 1a). Ten patients had pCR and nine patients had cCR after HT and TNT treatment, corresponding to a total CR rate (sum of pCR and cCR) of 39%. Details are shown in Table 3. Patients between 50–69 years represented the most frequent age group and showed the highest CR rates (45%, Fig. 1b). Moreover, a change in UICC stage was observed in 35 of 49 (71%) patients, most of them showing an improvement (32/35, 91%). At the end of therapy, 19 patients with CRs were staged UICC 0. Furthermore, UICC I was reached for nine patients, UICC II for eight patients, and UICC III for nine patients. Distinct oligometastases (UICC IV) were still diagnosed in four patients. Figure 1c shows the UICC stage distribution before and after therapy for all patients. Regarding the TNM classification, downstaging of the prognostically important cN category was observed in 36 patients with cN+ to 22 patients (61%) with ypN0 category.

**Table 3:** Response rates with 95% confidence interval versus historical reference.

Response	*n*/*N*	Rate (%)	95% CI (Wald) (%)	Reference (%)	Wald z	*p*
cCR	9/49	18	8–29	3	2.78	0.005
pCR	10/49	20	9–31	25	−0.80	0.425
CR (sum)	19/49	39	25–52	28	1.55	0.122

*cCR* clinical complete response, *pCR* pathological complete response

Subgroup analysis of patients with fewer than six deep HT sessions (less than once per week, *n* = 7/49) showed that in two of seven (29%) patients, CR (1 pCR and 1 cCR) was achieved. With prolonged HT treatment (defined as 6 or more HT sessions), CR was achieved in 17 of 42 (40%) patients.

**Fig. 2:**
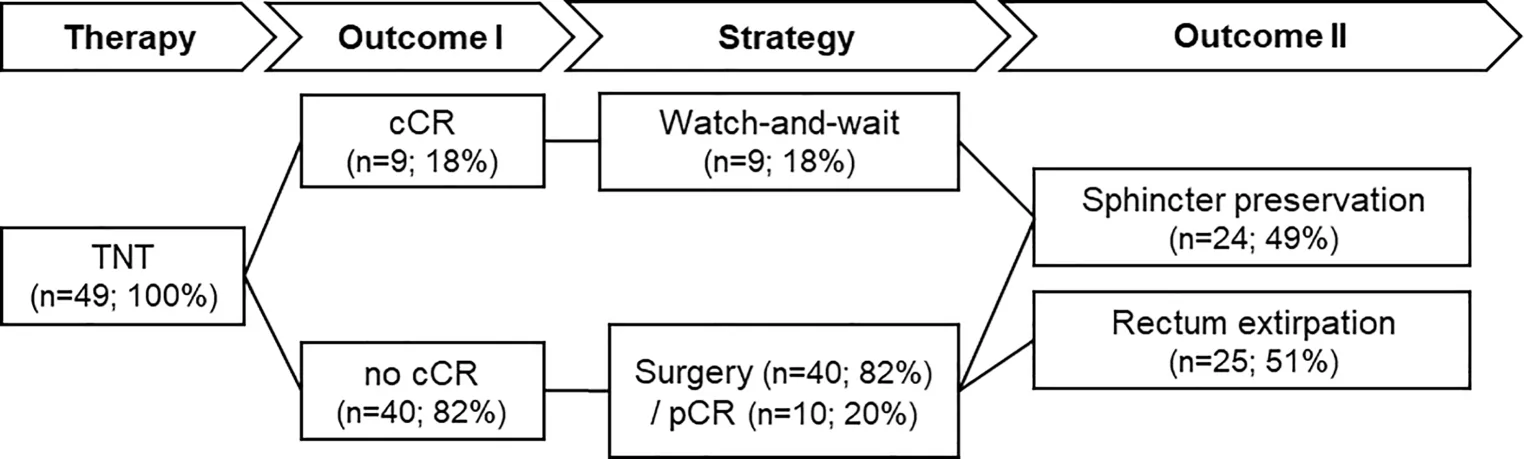
Flowchart for patients with rectum carcinoma and total neoadjuvant therapy (*TNT*) treatment. *cCR* clinical complete remission, *pCR* pathological complete remission

The patient treatment pathway is shown in Fig. 2. Nine of 49 patients (18%) achieved cCR and were managed using a “watch-and-wait” approach without further intervention, while 40 of 49 patients (82%) underwent subsequent surgery. As a result, sphincter preservation was achieved in 15 of 40 patients (38%), while 25 of 40 patients (63%) required total mesorectal excision/total rectum extirpation. Five of these patients were staged as pCR. Considering the patients without surgical intervention, sphincter preservation was achieved in 24 of 49 patients (49%).

Among the nine patients who achieved a clinical complete response (cCR), the estimated 3‑year DFS rate was 89% at a median follow-up of 41 months. At the latest follow-up, seven patients were still in sustained cCR.

Based on the hypothesis that higher mean HT temperature could improve the therapy outcome, the influence of the temperature during HT treatment on the outcome was analyzed. No significant differences in mean HT temperatures in terms of CR rate (*p* = 0.121) were observed. Interestingly, patients who had sphincter preservation (including patients with cCR) had significantly higher mean HT temperatures than did patients with rectum extirpation (*p* = 0.033).

## Discussion

Despite evidence supporting its effectiveness, HT remains infrequently used for rectal cancer treatment, even in the era of advanced multimodal therapies such as TNT. For decades, HT has been recognized for its potential to enhance the efficacy of radiotherapy and chemotherapy. Specifically, HT enhances the efficacy of treatments by increasing drug uptake and by inhibiting DNA repair mechanisms and heat-dependent immunological reactions, e.g., through heat-shock proteins and other apoptotic pathways [26, 27]. However, its integration into routine clinical practice has been limited, likely due to logistical challenges, insufficient clinical data, and lack of standardized protocols. This limited use highlights a significant gap in achieving the full therapeutic potential of HT, particularly in improving outcomes for patients with LARC.

For many years, HT in combination with radiotherapy and/or chemotherapy was not considered for neoadjuvant treatment. In oncology, the efficacy of HT was demonstrated for high-risk soft-tissue sarcomas in a randomized phase III trial, which showed that adding deep HT to standard chemotherapy was beneficial. This approach significantly and sustainably improved local control and OS in patients [23]. The R0 resection rate, a measure of local response, was also increased from 42% with neoadjuvant chemotherapy to 51% with HT [23].

In rectal cancer, the potential role of regional HT is increasing, as recently demonstrated in several clinical trials. A phase II study added regional HT to standard preoperative CRT, resulting in a high CR rate and improved long-term recurrence-free survival [24]. The present study demonstrated an overall CR rate of 39%, which is approximately 10% higher than the rate observed in a comparable approach without deep HT (ARO 04). Schroeder et al. [21] also demonstrated in their analysis that the addition of effective HT increases the rates of CR by approximately 10%. In both patient collectives, CR was associated with improved survival. Due to limited follow-up data, a correlation of increased CR rates with improved DFS and OS was not considered in our analysis.

In the TNT concept, the CAO/ARO/AIO-12 trial compared CRT plus induction or consolidation chemotherapy for LARC [18]. This trial is of special interest since the treatment regimen of arm B (additional HT) was comparable to the treatment applied in our study population. Taking into account the limited comparability between studies, the cCR rate (18%, 95% CI: 8–29%) significantly exceeded historical CAO/ARO/AIO-12 benchmarks (3%, *p* = 0.005), indicating superior clinical complete response. Overall CR rates showed numerical improvement (39% vs. 28%, *p* = 0.122), while pCR rates were comparable to controls (20% vs. 25%, *p* = 0.425). Moreover, observed pCR rates in our population were comparable to those from the UNICANCER-PRODIGE 23 trial (28%) that investigated neoadjuvant chemotherapy with FOLFIRINOX followed by chemoradiotherapy, surgery, and adjuvant chemotherapy in LARC [5]. Similarly, the RAPIDO trial evaluating short-course radiotherapy followed by chemotherapy before total mesorectal excision showed pCR rates of 28% in the experimental group, indicating the increased efficacy of preoperative chemotherapy in LARC [6]. Both trials showed lower frequency of cCR rates followed by watch-and-wait strategies (1% and 3%, respectively). Although our study primarily focused on early efficacy endpoints and survival data remain immature, patients achieving cCR demonstrated sustained disease-free status. These findings highlight the durable efficacy of TNT incorporating HT. However, these findings are based on a small cohort and should be interpreted with caution, as the data are preliminary and limited by the duration of follow-up.

The results of our study are consistent with other studies showing that the addition of HT can improve complete remission rates by about 10%. Furthermore, Gani et al. [28] and Ott et al. [19] showed the feasibility of HT, with high efficacy and comparably minimal acute and late toxicities.

Nevertheless, the addition of HT treatment leads to increased tumor regression and increases the rates of sphincter-preserving operations by about 20% [20]. The decision to preserve the sphincter using surgical techniques or to follow a watch-and-wait approach is challenging and depends on the surgeon’s expertise and mindset. Since five patients in our study had rectal extirpation after achieving pCR, it is important to discuss whether a watch-and-wait approach or surgery is more beneficial in terms of quality of life, recurrence, and OS. This requires careful, individual, case-by-case decision-making and should be evaluated in prospective studies.

All these studies suggest that HT, when combined with chemotherapy and radiation, may enhance treatment efficacy in rectal carcinoma. In a recently published systematic review and meta-analysis, including 12 studies and 760 cases of locally advanced rectal cancer, neoadjuvant CRT in combination with deep regional HT achieved a pCR rate of 19%. With classic neoadjuvant CRT using 5‑FU or capecitabine, pCR rates of approximately 10% can be achieved [29]; adding HT could thus increase pCR rates by approximately 10%. In conclusion, better response rates and higher downstaging rates are associated with a better prognosis [11].

Moreover, the frequency and mean temperature during HT may influence the therapy outcome in patients with LARC [28]. In patients with fewer than six HT sessions (*n* = 7), the CR rate was slightly reduced (29%); however, this is limited by the low number in our study population. Schroeder et al. [24] found that at least four HT treatments in combination with neoadjuvant RCT achieved a significantly increased pCR rate of 22.5% compared to treatment without HT (*p* = 0.043). Interestingly, our data showed that higher mean temperatures during HT treatment were associated with higher rates of sphincter preservation. The positive effect of higher HT temperatures on LARC outcomes is consistent with a study that showed improved recurrence-free survival rates depending on the HT treatment temperature [28]. It is suggested that higher cumulative thermal exposures in terms of frequency or mean temperature could improve patient outcomes. Nevertheless, the findings of our study should be interpreted with caution due to the limited sample size and the potential influence of outliers and confounding factors.

### Limitations

The present study has several important limitations. As a single-center retrospective analysis with a limited number of patients it is subjected to selection bias and limited external validity. The limited availability of long-term follow-up data on OS and DFS, as well as tumor regrowth rate, restricts the assessment of long-term outcomes. Furthermore, the variability among surgeons performing the procedures may have introduced inconsistencies in surgical technique and patient outcomes. Moreover, the absence of a standardized watch-and-wait protocol could influence the outcomes. Additionally, the limited availability of detailed tumor biology data, including molecular and genetic characteristics, may confine the identification of potential prognostic and predictive factors. This analysis was restricted to short-term toxicity, and late toxic events were not evaluated. Future analyses with prolonged follow-up data are needed in order to consider tumor regrowth, DFS, and OS as important clinical endpoints.

## Conclusion

This study shows the potential of deep hyperthermia (HT) in the treatment of locally advanced rectal cancer. The simultaneous application of HT to long-course chemoradiotherapy in total neoadjuvant therapy protocols is possible with manageable acute toxicity and increasing complete remission rates. Detailed data regarding survival parameters such as disease-free survival or overall survival are needed to evaluate the (long-term) efficacy. Future prospective studies are needed to confirm these results.

## Data Availability

The dataset generated and/or analyzed during the current study is available from the corresponding author upon reasonable request and after consultation with the local ethics committees.
